# On Lesions of the Cutaneous Sensibility among the Insane

**Published:** 1860-01-01

**Authors:** TH. Auzouy

**Affiliations:** Médecin-en-chef de la Division des Hommes à l'Asile public d'Alienés de Maréville


### Art. IV.—ON LESIONS OF THE CUTANEOUS SENSIBILITY AMONG THE INSANE.

By Dr. TH. AUZOUY, Médecin-en-chef de la Division des Hommes à l'Asile public d'Aliénés de Maréville.

(*Translated from the Annales Medico-Psychologiques.*)

Many causes combine to modify the physiological functions of the skin in persons of sane mind; but these alterations are infinitely more frequent and more important among the insane. This fact has often been incidentally remarked in reference to particular cases, but it is capable of much more general application than has been supposed. I have been convinced by experiments which I have perseveringly followed for a long time, that in the case of a large number of insane patients there exist functional disorders of the cutaneous organs, and that the most common of these disturbances is anæsthesia. This phenomenon so far as concerns idiots did not escape Esquirol. "Idiots," says he, "sometimes live under the utmost physical insensibility, although possessing their senses. Some of these unhappy beings have been known to bite and tear themselves, and to pluck out their own hair. I have seen one idiot who with his fingers and nails had pierced a hole in his cheek, play with a finger in the opening, and who ultimately tore it open as far as the commissure of the lips without appearing to suffer. There have been others who have had their feet frozen, without appearing to give the matter any attention." I have under my care at Maréville an idiot who amuses himself by running rough wooden pegs through his nose and ears, and who baffles the attention of those who would attempt to deprive him of this singular pastime.

It is far, however, from being the fact, that idiots alone possess the sad privilege of being insensible to pain. Long ago my honoured colleague and fellow-labourer M. Renaudin pointed out anæsthesia of the skin as furnishing the most certain elements of diagnosis in many instances of insanity. It was at his instigation that I patiently investigated this anomaly in the course of my attendance upon more than 600 invalids; and, to my great surprise, I found that more than half of them presented different degrees of analgesia of the cutaneous organ. This enormous proportion of analgesics will appear less extraordinary if it be considered what a large element of our population consists of the demented, idiot, imbecile and melancholy class.

One of our first physicians, M. Beau, has shown that pellagra and that form of analgesia which belonged to the *illuminati* or *convulsionaires* of past times, who imbibed from the exaltation of religious monomania an exclusive concentration of sentiment and an absolute impassibility with regard to the most cruel tortures, depend upon the sensorial lesion inherent in certain nervous disorders, such as hysteria, nervous delirium, and lypemania. M. Beau has also established a distinction as exact as it is ingenious, between anæsthesia in reference to pain (or *analgesia*), and anæsthesia in relation to feeling or sensation; he has justly remarked that tactile anæsthesia necessarily carries with it anæsthesia as to pain, but that the reciprocal proposition is not true. In fact, analgesia most frequently exists amongst the insane without the loss of tactile insensibility.

M. Michea has reported a series of observations which place beyond a doubt the existence of analgesia in the case of most persons afflicted with melancholia, and notably so amongst those suffering from religious and suicidal lypemania. With reference to this, M. Legrand du Saulle has quoted a remarkable case observed by him in the asylum at Dijon. An old man, called Mairet, sixty-one years of age, believed himself to have been dead for forty years, and besought that he might be buried. In order to ascertain whether his delirium would survive the accomplishment of his desire, he was literally buried up to the neck, and if he complained of anything, it was that his interment was not completed. This patient shortly afterwards received a serious wound, which did not cause him any pain; no more did the application of cupping glasses and various irritants, which he scarcely appeared to perceive.

A melancholy patient in the asylum of St. Yon, one day after having stabbed himself with a knife several times in the vain attempt to kill himself, thrust the prongs of a fork into his breast, and perceiving that the instrument was not placed quite opposite his heart, pulled it out with great coolness, and stuck it in again on a level with the left ventricle, which he fatally reached this time by a voluntary pressure, and by setting his body against the table on which his meal was being served.

A girl named Marie Jallot, in the asylum of Fains, eighteen years of age, one day eluding the watch kept over her, suddenly opened a stove which was red hot, rapidly thrust in her head, and fixed her chin so well against one of the sides, that it was only with great difficulty she was snatched from her voluntary punishment, of which she seemed scarcely to feel the consequences. This patient has survived her horrible burns; her delirium is in no way modified.

The Brussels newspapers lately reported the following fact, which was thought to be unparalleled.

A workman employed by one of the principal gold-beaters of the town, and named X——, had quitted his work before the end of the day, feeling, as he said, a very violent headache. Next day he left the workshop for the same reason, and returned home. There his suffering continued, but his condition presented no alarming symptoms. Suddenly, however, the unfortunate man sprang from his chair, and possessed by a horrible hallucination, threw himself upon the stove, which was then more than usually heated for some domestic purpose and almost thoroughly red hot; he embraced it with his arms, pressing it with all his force against his breast. The piercing screams of his wife, who endeavoured in vain to put an end to this fatal embrace, brought the neighbours to her assistance, and they succeeded ultimately in detaching the unhappy man from the stove, which he continued to clutch with the stoicism of a mad insensibility to physical suffering. But already the whole anterior portion of his body presented a frightful aspect, and fell off, so to speak, in rags, consumed by prolonged contact with the block of red-hot iron, which in like manner seared his arms and seamed them with horrible burns. At the end of a few hours X—— expired, in spite of the best medical assistance, and in the midst of unheard-of sufferings.

This example may be matched by the following, which was published in 1851, in *L'Union Médicale* by Dr. Morel, one of my honoured predecessors at Maréville.

A man still young had just celebrated his second marriage. In the midst of the preparations for the wedding-feast the bridegroom quitted the company, and after his prolonged absence had thrown the family into a state of some uneasiness, a search was made for him. On entering the nuptial chamber, the following spectacle presented itself to his friends and relatives. On a vast brazier lay a corpse half consumed, and the medico-legal examination which followed, verified the fact that the unhappy man, after having laid himself down on the fire, had preserved sufficient presence of mind to turn himself, and so render his combustion more complete.

The history of the shoemaker, Mathew Lovat, as reported by Marc, is pretty generally known. He commenced his long martyrdom by amputating his genital organs, which he threw out of the window. This horrible mutilation was scarcely healed when he crucified himself, after having made the most minute preparations for his torment, which he all but succeeded in consummating. With his feet and hands pierced with enormous nails, which he had obstinately thrust into them, his side laid open with a paring knife, he remained completely insensible during eight days, at the end of which period his capacity for pain returned.

The pathological anomaly of the general power of feeling has been pointed out by M. Renaudin as constituting the initiative phenomenon of monomania. Here, as in lypemania, the absence of feeling is not to be attributed so much to defective innervation as to the absorption of the sensitive faculty by the close and exclusive contemplation of some object which admits of no other mental action. MM. Morel and Renaudin have quoted in their works the interesting observation of the case of a man named Creut, who is still at Maréville, with a tendency to dementia. He is a monomaniac with extatical hallucinations; and, being ambitious of imitating the martyrdom of St. Lawrence, on the festival of this saint he plunged his right arm into boiling water, and opposed the efforts of the attendants to pull it out with a resistance almost tetanic. He remained completely insensible to pain until the day following this act of supreme folly. I meet with facts daily showing how difficult it is to act upon this man with any stimulant.

M. P——, a judge at V——, who was affected with religious monomania, devoted himself to the stake in order to expiate his sins, and after having himself constructed the pile and lighted it, he there burned himself until his fat trickled upon the stones, the bones of his members were calcined and whitened, and nearly his whole body carbonized. The countenance of the dying man when his physician arrived denoted a state of beatitude; he betrayed neither pain nor emotion.

With some maniacs as well as with monomaniacs nervous centralization supervenes to such a degree that the faculty of receiving external sensorial impressions disappears. The state of torpor as to external feeling, although analogous in its expression to that which manifests itself in persons who are stupid and deprived of their senses, emanates from a cause totally different. In the one case it results from the absorption of psychical activity by an exclusive class of ideas; in the other, the sensorial lesion must be imputed to a state of defective innervation. Here the nervous fluid, without losing any of its energy, is concentrated upon one special object; there, on the other hand, according to the happy expression of M. Renaudin, it produces *une véritable nevrorrhagia.*

Physical insensibility manifests itself sometimes in a transitory form, and exists only during the continuance of the paroxysms in certain maniacal fits. We have at Maréville a young patient who exhibits one of these deliriums of action, which only shows itself in an instinctive automatism without his having any delirious conception or incoherence either in his words or writings. Arthur D——, a youth of good talents, and till then tractable, became suddenly undisciplined and rebellious beyond measure; he gave himself up to the worst tendencies, and was on the point of imperilling his own honour and the repose of his family. He answers clearly, but with contrition and shame, that his perverse acts were dictated by an inclination stronger than his will, and that it was not in his power to act otherwise than he did. His appearance is such as to banish all idea of mental alienation, and a superficial examination would have led to his being treated as a bad character of the ordinary stamp ; but the investigation which was undertaken by my colleague brought to light a state of complete cutaneous insensibility, which was evidently the pathological key to the position. Since his admission to Maréville, Arthur has from time to time experienced several intermittent attacks of anæsthesia, the appearance of which invariably coincides with irresistible longings of the worst description, while the return of cutaneous sensibility is immediately followed by moral dispositions of a totally different character.

In some cases of mania, and of acute and nervous deliriums, it happens that anæsthesia of the skin is not accompanied by coldness of the periphery; but, on the contrary, there is an increase of caloricity. The eyes are brilliant and animated, the face flushed, the skin bathed in perspiration, and the patient greatly agitated. Invalids in this state are inaccessible to all physical pain. They have been known to lean with impunity upon members horribly fractured, to mutilate and tear their own flesh with pleasure. At the Fains Asylum I have reduced serious fractures in the cases of two lunatics afflicted with acute delirium; I have made deep incisions and sutures on the cranium of an oinomaniac without any of the patients appearing to be aware of the operation.

It is especially amongst individuals afflicted with stupor that the annihilation of general sensibility attains its climax. MM. Baillarger and Delaisauve have remarked this as one of the most constant symptoms. *Hands have they, and they handle not*, &c., may be truly said of melancholics whose mental faculties are completely torpid (*stupides*). The greatest obtuseness takes possession of their sensations as of their sentiments. Lypemania, adds our colleague of Bicêtre, by the aggravation of its determining lesion, is apt to lead to torpor, and sensational paralysis is necessarily excited by the prostration attendant upon lypemania. On the other hand, when monomaniacs and lypemaniacs pass to a state of dementia they show themselves less tenacious of their opinions and less obstinate in their silence; they recover a factitious animation, and in the midst of a morbid condition more alarming in character, they put on outwardly a deceitful amelioration. These invalids, in fact, recover, with the disappearance of nervous tension, a certain amount of purely ephemeral sensibility; for their external impressions are not slow to become confused, and as the nervous system becomes weaker, cutaneous anæsthesia sets in again and progresses indefinitely. The result of my own observation is, that in the case of demented persons, imbeciles, and idiots, the lesion of the general sensibility is with very few exceptions in direct proportion to the lesion of the intellectual faculties. The tegumentary envelope of these weakened and depressed beings, in whom the physical and moral faculties verge equally torwards torpor and inertia, and in whom spontaneity tends gradually to vanish, participates passively in the general cachexy of the organism.

Notwithstanding, however, that perceptive sensibility is so often diminished or destroyed during madness, it is sometimes greatly increased. Cases of hyper-æsthesia are met with especially amongst maniacs and lypemaniacs labouring under hallucinations. The sufferings of these last, though imaginary, are none the less felt with extreme acuteness. There are beings with such impressionable organizations that the slightest external agent produces on them the most intense and painful effects: to them the wind is always in the east. I once knew a lady who persistently avoided walking in the open air, on the sole ground that the leaves falling upon her from the trees caused her such frightful bruises and such atrocious pain, that she preferred death to it. On the other hand, some idiots experience vivid sensations of enjoyment when they are touched or lightly caressed on the nape of the neck. They have retained an aptitude for the sense of pleasure as well as pain, and are excessively pusillanimous. Fear or the sight of suffering suffices to draw from them plaintive cries and torrents of tears. Hypochondriacs experience visceral hyper-æsthesia produced by the increased energy of the ganglionic nervous system. The great sympathetic in them, indeed, acquires an increase of sensibility in proportion to the diminution which takes place in that of the cerebro-spinal system. . . .

Whenever I examine a patient, I endeavour to ascertain the degree of physical sensibility which he possesses. This examination, as might easily be anticipated, has naturally led me to vary the means of exploration. In order that my experiments should be devoid of all inhumanity in their character, and not calculated to alarm the lunatic, I have sought carefully for stimulating agents of such a nature that they might also be available in the treatment of the malady. I hasten to declare that although the application of blisters, moxas, setons, the actual cautery, the use of cupping, &c., has occasionally furnished me with useful data by which to judge of the cutaneous activity, I have used these means with great reserve; never prescribing their application but in such cases where the special pathological condition imperatively called for their use. Punctures and pinchings, practised either with the hand or with blunted pincers curbed so that they cannot be perfectly closed, resemble too much means of torture to be employed except very rarely. Cold affusions, sudden besprinkling with a water-can, or garden-pump syringe, hydrotherapeutics applied without especial apparatus and according to the resources which I had at command, frictions with stimulating applications or with congealed snow, have been frequently and very advantageously made use of. I have used largely muscular exercise and manual labour, which at one and the same time stimulate the inertia and moderate hyperexcitation. At my request a gymnasium has been formed at Maréville for young subjects, and for those adults whose age or certain special conditions debar from the labours of the wood-yard or the workshop. This institution is certainly efficacious in reanimating the benumbed functions of the tegumentary system. Lastly, I have had recourse to urtication in some cases of stupor and excessive torpidity, in order to recal to the cutaneous surface its lacking activity; and I must say that the different means which precede, singly insufficient, can by a gradual and rationally conducted combination, lead to very satisfactory results. Nevertheless it is not sufficient to arouse solely a certain degree of sensibility in the periphery; it is requisite also to combat the fixed ideas of monomaniacs and melancholics, to break the exclusive concentration of their ideas, to reanimate the deadened intelligence of the torpid, and to give somewhat of elasticity and energy to the mind of the demented. In order to attain this end there are still other means than those which I have already enumerated. M. Moreau (de Tours) has extolled and tried with success the internal administration of hachish in analogous cases; but not having for the moment at my command the extract of Indian hemp, I have been obliged to postpone its administration to my patients. I have had recourse to anæsthetic agents, previously successfully experimented with at Maréville, by M. Morel, especially in reference to diagnosis and legal medicine. They ought to render me, and have rendered me, indeed, very great services. To use artificial anæsthesia with an analgesic may seem at first paradoxical; in examining, however, the mode of action of anæsthetic inhalations, we recognise that it exhibits several different periods, of which it is possible to profit according to the indications which we seek to satisfy. To arrest the inhalation as soon as the period of excitement has arrived, and to maintain for a certain time this physiological effect, such is the end that we ought to propose generally for the group of depressed patients in whom psychical force is wanting. It is thus that I act with regard to subjects attacked with inertia and stupor. The action of chloroform being very rapid and inducing very promptly the period of resolution, it becomes difficult with this substance to prolong without danger the excitation. In consequence of this I prefer æther. Ætherization may be prolonged with impunity during a period of time sufficient for a very marked effect to be produced upon the subject. The sole precaution required, in order to avoid overstepping the limit of excitation, is to suffer the patient to breathe the air, the inhalation of the æther being continued at intervals, in order to prolong its effects.

Pushed until the period of resolution and complete insensibility, ætherization occasions muscular fatigue and a favourable stupefaction, notwithstanding the reaction which succeeds them, in individuals who are affected with acute delirium accompanied with sleeplessness and uncontrollable agitation. If the first essays do not always induce the quietude desired, it is rare, if we persist, that we do not finish by obtaining more or less marked tranquillity. One of the most remarkable results that æthereal inhalations have given to us has been the transformation of delirium, which often changes its type and character in consequence of their employ. The enfeeblement of the mental malady, or the substitution for it of a form more accessible to the ordinary means of treatment, would be assuredly a great benefit gained. But better still than this, we owe to the use of artificial anæsthesia many cases of cure. . . . .

Among our more energetic therapeutical agents it is of importance to choose one which permits us in some sort to dose the disorders of innervation among the insane, to measure the different degrees of their sensibility, which can, in fact, serve us as a sensitive thermometer. [This means of investigation is at hand in the form of electricity, and the mode of applying this force, and the results obtained from its use at Maréville are as follows] :—

Our patients are, for experiment, fixed in an arm-chair in such a manner as may be requisite, and placed in front of a little table upon which rests the electro-magnetic machine. Nothing is easier than to submit them to the action of a current by means of metallic conductors, sometimes furnished with humid sponges, and held by the operator by means of non-conducting handles. I shall now sum up as accurately as possible the facts that M. Renaudin and myself have demonstrated in our different experiments.

M. Renaudin has accumulated in the female division of the asylum observations respecting six cretinous idiots, eighteen idiots, and thirty-two imbeciles; total, fifty-seven patients.

*Cretinous Idiots.*—Of the seven cretins, in four there was no sensibility whatever, and the strongest electric current did not disturb their impassibility; in two, the skin had a very obtuse sensibility, appearing to be affected feebly, and the manner in which they supported themselves indicated how confused was the sensation which they experienced; in the seventh, the sensibility was nearly normal, the electric shock being very sharply felt.

*Idiots.*—Experiments were made upon eighteen patients of this category. In four of them the skin was every way insensible; submitted to the maximum intensity of the electro-magnetic action, these women did not suffer from any painful sensation, but solely exhibited very lively muscular contractions; in seven others the skin was arid and dry, but preserved traces of obtuse sensibility. In these subjects the electric fluid produced tolerably energetic contractions and a sensation which was made manifest by groans, but which was evidently less intense than it ought to have been, because the perception of it was imperfect; in five of these patients, the skin had maintained its normal suppleness and impressionability: the electric shock was felt with vivacity. Lastly, in two others the skin was very impressible, being unusually sensitive to the electric current. The shock extorted from them tears and piercing cries.

*Imbeciles.*—The observations made and repeated upon thirty-two of them led to their being subdivided in the following manner:—Five patients, in whom the skin was absolutely insensible to punctures, pinches, and to the most energetic stimulants, underwent but slight contractions, although not the least painful sensation, when the maximum current acted upon it; nine had scarcely a remnant of aptitude to feel exterior agents, and the current, of which the strong shock agitated them a little, caused them rather surprise than pain; nine others, who felt but confusedly, were impressed by fluid. This impression, in other respects obtuse and vague, vanished without leaving a trace. Seven had normal sensibility in the periphery; hence the electro-magnetic current acted energetically; the contractions were strong, hasty, and painful. Two, lastly, were pusillanimous in the last degree; the current terrified them and extorted from them the most bitter groans.

I have submitted to the influence of the electro-magnetic current about the fourth of the population in the male division of the asylum. 150 lunatics, taken indiscriminately, have been submitted to repeated experiments, of which the results have been noted exactly.

*Cretins.*—Two cretins in our wards are entirely insensible to exterior stimulants, whatever be their energy. The maximum of the current excited in them but slight contractions, accompanied by an air of stupid hilarity.

*Idiots.*—Of nineteen idiots who were electrified, nine did not feel any shock, and were even quite unconscious of the operation. Their cutaneous sensibility was absolutely nothing: a seton was applied to them, as one would stitch a mattress. In four, the tactile sensibility was very obtuse, scarcely affected by the action of fluid; four others retained a vestige of physical sensibility, and were moderately impressible; and lastly, there were two in whom the sensitiveness of the skin was nearly normal, and who uttered acute cries when the current acted upon them.

*Imbeciles.*—Twenty-six imbeciles were electrified. Three of them, anæsthetic in the highest degree, were with regard to the fluid, as the most degraded cretins and idiots; eight, very nearly insensible, were very slightly aroused, and gazed on all sides, more affected by the unaccustomed apparatus displayed around them, than with the effect it produced upon them. There were six in whom the deadened sensations were feebly awakened by electrization, which caused the muscles to contract, and provoked astonishment, rather than pain. Seven imbeciles had some aptitude to feel external stimulants; the electricity caused them to flush, and at the end of some instants' application it extorted from them complaints. Finally, there were two extremely impressionable, upon whom the current produced instantly pain, which was shown by tears, and the most violent cries.

*Epileptics.*—From what precedes, we see, that a true gradation is observed in the manner in which idiots and imbeciles of both sexes feel pain. The electric agent measures, with a degree of exactitude at which we could never have arrived without its aid, the physical sensibility of each subject. That which is most striking in our experiments, is that the accessibility, and the suffering provoked, coincide with the moral aptitude, and that electrization acts more or less energetically upon the sensibility according as the individual is besides endowed with the faculty of forming his ideas more or less clearly, according as he is more degraded in the intellectual scale, or more perfect in his psychical aptitudes. The rare exceptions that we have met with to this rule, have all been idiots or imbeciles, who have been epileptic. This redoubtable neurosis places them in pathological conditions so abnormal, that I have thought it my duty to be extremely reserved in regard to them, and I have not continued to submit epileptics to electrization.

*Demented.*—Twenty-nine of the demented were discovered to be anæsthetic in various degrees. Electricity constantly had upon them an action in relation with their sensitive aptitude; six felt no more the operation, than if other individuals than themselves had been operated upon; nine felt very feebly: the contractions were lively, but nothing indicated a painful sensation; six were accessible to the electric influx, but in a very limited manner; they flushed easily. Five were impressed in a very real but transitory fashion; the experiment finished, they lost immediately the remembrance of it. Lastly, three were vigorously shocked, uttering cries, and weeping. I remarked that among these patients, the demented paralytics were the least accessible to pain, but that they were also those who flushed the most easily. The patients attacked with consecutive dementia were much more refractory to the current than those affected with primary dementia. It is among these last that we ought to rank those that the shock impressed with some vivacity. We noted also, that the destructives were all anæsthetic, and that, when the cutaneous sensibility reappeared, their destructive propensities ceased.

*Lunatics in a State of Stupor.*—Nine torpid lunatics appeared to us to be deprived of all tegumentary sensibility. They all, at first, maintained a complete impassibility to the exciting agents.

It was requisite to persevere in the experiments upon these patients. Two even to the end were rebellious towards an agent which hitherto has proved powerless in respect to them, but which, in three others, has momentarily awakened a little activity and overcome an obstinate mutism. In two lypemaniacs struck with stupor, our success has been more decisive; the induction shock has gradually reanimated the physical sensibility and markedly ameliorated the patient's condition. Two other cases have been indebted to electrization for a prompt cure.

*Melancholics.*—In the seventeen which I have observed, there were seven of whom the cutaneous sensibility was quasi-normal, and that faradization succeeded so far as to overcome voluntary mutism and obstinate refusal of food. In the ten others, the cutaneous sensibility diminished progressively; four felt freely the shock; the six others were in the highest degree analgesic and impassible to excitation. Their muscular contractions were always energetic.

*Monomaniacs.*—Thirteen monomaniacs, examined with care, furnished six cases of well-marked analgesia. In nearly the whole of the others there existed, in different degrees, a diminution of sensitive aptitude. The action of the pile, insignificant at the beginning, in the six analgesics became more active in them in proportion as the experiments were prolonged and repeated. Four, in whom the sensibility is variable, were greatly disturbed, and the three last were susceptible in the highest degree. The greater portion professed a deep aversion to the operation that they underwent; they protested and declared that it was supernatural and illegal. Three of these lunatics, under the influence of the current and the pain which it determined, renounced plainly their delirious ideas; they were momentarily reclaimed to a feeling of reality, and they abdicated their chimeras and gave true and conclusive explanations of their situation. Although transitory, this result is not the less important, and it permits us to hope for more desirable results in those subjects in whom the idiosyncrasy is less rebellious and the delirium less inveterate.

*Maniacs.*—The maniacs are, of all the insane, those who appear the least likely to derive any benefit from an electrical treatment. I have, nevertheless, applied it to thirty-five of these cases, and I confess to have been little encouraged to reiterate often my essays upon them. Patients suffering from chronic or remittent mania, preserving their nearly normal sensibility, are sharply agitated and disturbed by the passage of currents. Some, indeed, struggled and upset the apparatus, vociferating and even defæcating involuntarily during the operation. In their paroxysms they feel no pain, it is true, but their muscular contractions are so hasty and disordered that their agitation is rather increased than diminished. This result need not surprise, if what I have said respecting the action of electricity in other forms of insanity be remembered. We have seen it suspend momentarily the delirious conception of the monomaniac, in modifying, for an instant, that nervoso-cerebral concentration which is peculiar to these lunatics; we have aroused the cerebral activity of the lypemaniac, of whom all the sensibility had been concentrated in the ganglionic nervous system. Lastly, we have succeeded in reanimating the vital energy of the demented, in whom the innervation is incomplete; but do we encounter in the maniac any one of these indications? Evidently not. Here, there is no concentration in one sense or the other; there is a convulsive state more or less permanent, and that which I have said of the action of electricity in epilepsy explains sufficiently why this means is inefficacious and even noxious in acute mania, and why it leads to few results in chronic mania.

I cannot then admit that mania may be cured by electrization, neither can I admit that electricity is a simple and commodious means of coercion, and that it may advantageously replace the camisole, the douche, &c. The manipulation of an inductive apparatus presents difficulties and exacts precautions which render necessary the constant intervention of the physician in their application. Admitting the hypothesis even that sedative effects may be obtained from its use, these would be too transitory, too fugacious, to dispense with the use of the camisole and the ordinary soothing means, of which the principal inconvenience, in my opinion, consists solely in the abuse to which they are liable, and which has happened sometimes. . . . .

I believe that I have sufficiently shown in the course of this paper, that analgesia is a pathological state which constitutes not solely a fortuitous event peculiar to some cases of mental alienation, but rather a very frequent symptom of which the appearance is intimately bound with the generality of the types of insanity.

This immunity from pain, independent of the alterations of which the sense of touch may itself be the object, is witnessed in various conditions, according to the form of delirium that it accompanies; it is in general proportioned to the moral lesion, increases or decreases with it, and influences powerfully the development and progress of the diseases incident to the insane. The history of mental alienation furnishes numerous examples of this physiological and pathological modification which had given rise to a prejudice long fatal to the insane; because, if it were an epoch when their clothing was neglected, or they were left to stagnate in cold and damp huts, and when their nourishment in the hospices was the remnants of that which had been served to the rational infirm, those who were reproached for these culpable neglects answered then that lunatics felt nothing. But, if they felt not the impression, it is rare that they did not feel the effects, especially when this anæsthesia was the result less of a disorder of the sensibility, as the expression of a notable diminution of the vital energy.

I have then had principally at heart to prove that the practitioner ought to regard analgesia seriously, and not to neglect any of the agents capable of modifying the general sensibility. It was to this end that I undertook, with the intelligent assistance of my *internes*, and especially of M. Kuhn and Dr. Schællhammer, my researches upon ætherization and electrization of the insane. They are far from having been sterile: if they have not yielded, in a curative point of view, all the results that it is possible to attain, at least those we have obtained are not to be disdained. The action of the electro-magnetic current, always inoffensive and exempt from danger in its application, contributes in a most efficacious manner to reinstate the sensibility in that place where it is wanting, and to give elasticity and energy to the deadened muscular system. This medication, new in mental alienation, is not then rash; perhaps even subsequent essays, carried out by hands more able than ours, will ultimately prove that we have not attributed to it all the therapeutical value that it merits.

An essential point with us was the power to demonstrate with some precision the degree of anæsthesia, of pain or of analgesia, that lunatics present in the course of their mental affections. Has this end been attained? The affirmative cannot be doubted, because we seek vainly a physical agent which permits us to appreciate with more exactitude the degree, and the kind of sensorial lesion of each individual. The electrical influence being in direct ratio of the exterior sensibility, and of the intellectual development of the subject, whatever be besides the particular type of his madness, it results that the electricity of induction can be considered as a precious means of diagnosis. This element of exploration, common now at Maréville, is then fitted to render useful services to mental pathology.

As a therapeutical agent, it is especially with a view to impress upon the economy a salutary perturbation that I have used electrization. In the case where the madness is accompanied by depression, when it is manifested with apathy or stupor, electrization becomes sometimes, in the hands of the practitioner, an heroic remedy. It communicates to the nervous system a dose of activity which, although factitious, accelerates the circulation, and favours the functional activity of the cutaneous system. It serves advantageously to vanquish resistances which it is important to overcome, such as the refusal of food, voluntary mutism, inertia, &c. With its help, we can suspend momentarily the delirious conceptions, and even bring about little by little their suppression.

I am convinced that cataleptics, so refractory to all stimulants and external agents, would undergo happy modifications if they were submitted to an electro-magnetic treatment. Catalepsy is so rare an affection, that I have not yet had an opportunity of treating it with electrization.

The legal medicine of the insane it seems to me ought also to derive some advantage from this source. If we have been able by the aid of ætherization to unmask the feint of individuals who simulate folly, with greater reason may we anticipate, by the means of electrization, to recognise frauds of this nature. The individual who is subjected to an energetic current cannot dissimulate that which he undergoes ; a force superior to the most tenacious will obliges him to throw off the mask and reveal himself such as he is. Electro-magnetic testing has powerfully seconded my investigations, when I have had to ascertain the mental state of a young conscript placed under observation in the asylum, and whose alleged imbecility was rightly doubted.

In that which refers to the anæsthetic action of electricity, our observations do not throw any light upon a question lately raised by eminent practitioners. The minor operations are ordinarily effected without pain upon lunatics, on account of the spontaneous anæsthesis which exists in the most part of them. We have not then experimented with faradization in reference to the deadening or suppression of physical suffering. The effects of stimulation and of excitation are the sole that we have at present examined, and that we have obtained, in the application of electricity to mental medicine. In treating here of the principal questions relative to the intervention of electricity in the medicine of the insane, my intention has been particularly to call the attention of my brethren to some important facts, of which the study has appeared to me too much neglected, whilst, on the contrary, the other branches of the curative art have found in electricity an efficacious auxiliary. This is the reason why I have sought to investigate solely the actions of this agent in reference to mental alienation, and to prepare the elements of an ulterior experimental research; never forgetting that if it be useful to make known the good results produced by the electric current, it is not the less necessary to guard against thoughtless infatuation.

---

### Art. V.—WILLIAM CULLEN:—A PSYCHOLOGICAL STUDY.*

